# Beliefs and performances of elementary school students to prevent road traffic injuries, using Health Belief Model: a study from Hamadan, Iran

**DOI:** 10.5249/jivr.v11i2.974

**Published:** 2019-07

**Authors:** Maria Ebrahimikhah, Abbas Moghimbeigi, Seyed Mohammad Mahdi Hazavehei, Forouzan Rezapur-Shahkolai

**Affiliations:** ^*a*^ Department of Public Health, School of Public Health, Hamadan University of Medical Sciences, Hamadan, Iran.; ^*b*^ Department of Biostatistics, School of Public Health, Hamadan University of Medical Sciences, Hamadan, Iran.; ^*c*^ Modeling of Noncommunicable Diseases Research Center, Hamadan University of Medical Sciences, Hamadan, Iran.; ^*d*^ Research Center for Health Sciences, Hamadan University of Medical Sciences, Hamadan, Iran.; ^*e*^ Social Determinants of Health Research Center, Hamadan University of Medical Sciences, Hamadan, Iran.

**Keywords:** Health education, School health promotion, Students' injury prevention

## Abstract

**Background:**

Road traffic injuries (RTIs) are important health problems and increasing knowledge on their prevention-related issues can be credible. This study aims to assess beliefs and performances of students to prevent road traffic injuries and their related factors, using Health Belief Model (HBM).

**Methods:**

This cross-sectional study carried out on a random sample of 500 fourth and fifth grades students of elementary schools in Hamadan city, west of Iran. The data gathering tool was a self-administered questionnaire designed on the basis of HBM constructs and also the knowledge and performance of the students in relation to prevent RTIs. To increase the accuracy of this study, the students’ road-crossing behaviors were observed in a simulated street in the school, using an observation checklist. Data were analyzed by SPSS 16.

**Results:**

The mean age of the participants was 10.51±0.50. About preventing RTIs, the mean scores of the students’ knowledge was 64.139, and regarding HBM constructs, the mean scores of their perceived susceptibility, perceived severity, perceived benefits, perceived barriers and self-efficacy were 82.817, 82.453, 82.451, 89.917, 84.343 and 91.250, respectively. The mean score of the students’ self-reported performances about traffic injury prevention was 48.750 and the mean score of their observed road-crossing behavior in the simulated street was 45.000. The final model of multiple linear regressions showed that the students’ sex (p=0.001), their knowledge (p less than 0.001), perceived susceptibility (p=0.002), perceived barriers (p=0.032), self-efficacy (p=0.001), and their observed road-crossing behaviors (p=0.019) predict the students’ self-reported injury prevention performances.

**Conclusions:**

Regarding prevention of RTIs, knowledge and performance of the studied students are undesirable. The study findings can help designing more appropriate prevention programs for them.

## Introduction

The total number of road traffic injuries (RTIs) is increasing steadily in the world, reaching 1.35 million in 2016.^1^ About 93% of the world's fatalities on the roads occur in low- and middle-income countries.1 RTIs are the important causes of mortality among the children and young adult aged 5-29 years.^1,2^ More than half of all fatal traffic injuries occur among vulnerable road users including pedestrians and riders, while in high income countries drivers constitute the greatest number of the victims.^1-4^

There have been found more than 260000 deaths and 10 million RTIs among children and youth aged 0-19 years, in 2004.^5,6^ As RTIs are the important cause of death, to teach the skills for crossing streets safely can be one of the important program to prevent these injuries.^7^ In Iran, the traffic injuries are the second cause of death after cardiovascular disease.^8^ As RTIs are a serious public health issue, their prevention demand coordinated efforts.^2^ In Iran, pedestrians are one of the most vulnerable groups to RTIs and their greatest contribution belongs to the children and students in early years of schooling.^9,10,11^ Children are considered to be the vulnerable group due to their physical, psychological and behavioral features.^12,13^ The continuation of the current trend of increasing fatal traffic injuries among children and lack of suitable preventive measures can be a great risk for social life. The best cost-effective method for reducing traffic injuries is to apply educational and preventive methods. In fact, the people’s attitudes, behaviors and lifestyles must be changed through spreading results of related studies.^14,15^ It must be noted that using theory and model, increases the likely effect of health education programs and helps identifying individual and environmental factors influencing behavior. Theories and models play role in designing comprehensive programs and their assessments and help the executive interventions achieve their goals in populations that no experiments have been conducted on them.^15^ The Health Belief Model (HBM) tries to explain health behaviors. Therefore, it focuses on attitudes and beliefs, including perceived susceptibility (beliefs about the likelihood of getting a disease or condition); perceived severity (feelings about the seriousness of contracting an illness or of leaving it untreated); perceived benefits (beliefs regarding perceived benefits of the various available actions for reducing the disease threat); perceived barriers (the potential negative aspects of a particular health action); cues to action (strategies to activate “readiness”), and self-efficacy (confidence in one’s ability to take action). Besides, this model is used to determine the relationship between individuals' beliefs and their health behaviors.^16^ In fact, this model helps knowing the behaviors, identifying the points in which the behavior must be changed towards preventive behavior and facilitating decision-making.^16,17,18^ This study aims to determine beliefs and performances of the students to prevent RTIs and also the related factors using HBM.

## Methods 

**Study design and participants**

This research is a cross-sectional study carried out on the fourth and fifth grade students of elementary schools of Hamadan city, west of Iran, during November and December of 2012. As the total number of elementary schools was 143, systematic sampling was used to increase accuracy of selecting schools. (Boys’ and girls’ schools had been individually identified on the map from north to south. Having chosen the first school, wechose 15 other schools by specified distance). After choosing 5 girls’ schools (four public schools and one private school) and 5 boys' schools (4 public schools and one private school), the number of 500 students were chosen from the fourth and fifth grades of these schools. The study procedure and data privacy were explained for the participants and their parents. Then the participants agreed to join the study after their parents’ permission letters were received. (response rate was 100%). 

**Data collection instruments**

A questionnaire was used to collect data from students. It consisted of four sections: 1. demographic (sex, school grade, household size, parents’ education, having parents with vehicles, commuting and having road traffic injury experience) in 18 questions, 2. performances in 10 multi-choice questions with the score ranged from 0 to 10, 3. knowledge in 13 multi-choice questions with the score ranged from 0 to 13. Getting the highest score showed the greatest knowledge of the students regarding to prevent of RTIs. 4. The constructs of HBM included perceived susceptibility in 4 questions, perceived severity in 5 questions, perceived benefits in 4 questions, perceived barriers in 7 questions, cues to action in 7 questions and self-efficacy in 4 questions, A 3-point Likert scale (completely agreed, agreed to some extent, disagreed) was used approach to scaling the responses of all of the constructs, except for cues to action. The score ranged from 4 to 12. Finally, cues for action had 7 multiple-choice questions about road and pedestrian safety training which students could choose more than one choice. The scores of each construct of the model were calculated out of 100 points for all samples. Then the mean of these scores were calculated for each construct.

It is necessary to mention that the assessment of preventive performance was carried out through self-report questionnaire. To increase the accuracy of this study, the students’ road-crossing behaviors were observed through crossing the simulated street in the schools, using observation checklist with five items which were all about manners of students’ road-crossing behavior. The validity of the questionnaire was assessed through getting points of views of health education and safety promotion experts. To assess the reliability, the questionnaire was completed by 30 students and kuder-Richardson and Cronbach's alpha coefficient were used. Health education experts were asked to help assessing validity of the questionnaire and Cronbach's alpha was used to measure the reliability of the questionnaire. Cronbach’s alpha was calculated to be 0.61, and 0.7 for knowledge and other items, respectively. The questions related to Health Belief Model constructs and their proportion for correct answers/mean (SD) which were scaled by Likert scale, have been shown in Appendix 1  and the observation checklist has been shown in Appendix 2.

**Appendix 1:** Questions related to Health Belief Model constructs and their proportion for correct answers/mean (SD) for Likert scale answers

Constructs	Questions(Scores)	Proportion/Mean(SD)*
**Self-reported performances**	**1. How do you usually cross the street?**	0.376
	I stand at the crosswalk. At first I look at the left side and then check the right side (1)	
	I stand next to the street, first I look at the left side and then check the right side (0)	
	I stand at the crosswalk, first I look at the right side and then check the left side (0)	
	I stand next to the street, first I look at the right side and then check the left side (0)	
	**2. Do you sit in the backseat of the car? ** Always (1) Sometimes (0) Never (0)	0.406
	**3. Do you usually wear a seatbelt when you are getting on a car?** Always (1) Sometimes (0) Never (0)	0.538
	4. From which following parts do you usually cross the street?	0.430
	Wherever that I can (0) Between the cars (0) From a footbridge or a crosswalk (1)	
	**5. Do you usually wear helmet, when riding on the back of motorcycle with the adults?** Always (1) Sometimes (0) Never (0)	0.727
	**6. Do you usually wear helmet, when riding a bike?** Always (1) Sometimes (0) Never (0)	0.403
	**7. If you want to cross the street and the pedestrian traffic light is red, what do you do?**	0.570
	I stop so that the cars pass the street (1)	
	I walk across the street with caution (0)	
	None of them (0)	
	**8. If you want to cross the street and the pedestrian traffic light is green, what do you do?**	0.586
	I stop so that the cars pass the street (1)	
	I cross the street quickly (0)	
	I walk across the street with caution (0)	
	None of them (0)	
	**9- If you want to cross the street and the car traffic light is red, what do you do?**	0.538
	I stop so that the cars pass the street I stop so that the cars pass the street (1)	
	I cross the street quickly (0)	
	I walk across the street with caution (0)	
	None of them (0)	
	**10- If you want to cross the street and the car traffic light is green, what do you do? **	0.540
	I stop so that the cars pass the street (1)	
	I cross the street quickly (0)	
	I walk across the street with caution (0)	
	None of them (0)	
**Knowledge**	**1. What traffic lights are installed on the crossroad of the streets?**	0.586
	Pedestrian traffic light (0)	
	Car traffic light (0)	
	Both the pedestrian and the car traffic light (1)	
	No lights are installed (0)	
	**2. What lights should vehicles pay attention to, when passing the street?**	0.418
	Pedestrian traffic light (0)	
	Car traffic light (1)	
	Both the pedestrian and the car traffic light (0)	
	None of them (0)	
	**3. What lights should pedestrians pay attention to, when crossing the street?**	0.512
	Pedestrian traffic light (1)	
	Car traffic light (0)	
	Both the pedestrian and the car traffic light (0)	
	None of them (0)	
	**4. What does the color "green" mean on pedestrian traffic light, for you as a pedestrian?**	0.862
	Stop (0)	
	Move (1)	
	Caution (0)	
	None of them (0)	
	**5. What does the color" yellow" mean on pedestrian traffic light, for you as a pedestrian?**	0.870
	Stop (0)	
	Move (0)	
	Caution (1)	
	None of them (0)	
	**6. What does the color "red" mean on pedestrian traffic light, for you as a pedestrian?**	
	Stop (1)	
	Move (0)	
	Caution (0)	
	None of them (0)	
	**7. Which of the followings have the right to use the sidewalk?**	0.852
	Just pedestrians (1)	
	Pedestrians and bicyclists (0)	
	Bicyclists and motorcyclists (0)	
	All vehicles (0)	
	**8. To cross the street, I should use,…**	0-614
	Footbridge (0)	
	Crosswalk (0)	
	Police or parent’ help (0)	
	All of three points are correct (0)	
	**9. For getting on which vehicle should I wear a helmet?**	0.622
	Bike (0) Motorcycle (0) Car (0) Bike and motorcycle (1)	
	**10. When riding or getting on which vehicle, should I wear a seatbelt?**	0.718
	Bike (0)	
	Motorcycle (0)	
	Car (1)	
	Bike and motorcycle (0)	
	**11. Which of the followings can be the reason of children’s injury while getting on a car?**	0.934
	Sitting at the front seat of the car (0)	
	Sitting on the backseat of the car and not wearing seatbelt (0)	
	Standing inside the car and sticking their head out of the car window (0)	
	All three items (1)	
	**12. Which of the followings should be done when crossing the street?**	
	First look at right side then left side (0)	
	Just look at right side (0)	
	Just look at left side (0)	
	First look at left side then right side (1)	
	**13. At what age can children sit at the front seat of the car?**	
	Over 6 years old (0)	
	Over 9 years old (0)	
	Over 12 years old (1)	
	Over 15 years old (0)	
**Perceived susceptibility**	**1. It is likely that when I cross the street, a car hits me and I suffer damage. **	0.682
	I quite agree (3)	
	I agree to some extent (2)	
	I disagree (1)	
	**2. If I neglect safety tips when getting on a car, it may harm me.**	0.388
	I quite agree (3)	
	I agree to some extent (2)	
	I disagree (1)	
	**3. If I neglect safety tips when riding a bike or motorcycle, it may be harmful to me.**	0.300
	I quite agree (3)	
	I agree to some extent (2)	
	I disagree (1)	
	**4. If I do not take caution when crossing the street, it may harm me.**	2.136 (0.760)
	I quite agree (3)	2.480 (0.734)
	I agree to some extent (2)	2.640 (0.625)
	I disagree (1)	2.682 (0.591)
**Perceived severity**	**1. My injury may be harmful to my health. **	2.524 (0.668)
	I quite agree (3) I agree to some extent (2) I disagree (1)	
****	**2. My injury can damage my appearance and beauty. **	2.426 (0-685)
	I quite agree I agree to some extent (2) I disagree (1)	
	**3. My injury can cause damage to my studying and my daily tasks. **	2.456 (0.681)
	I quite agree (3) I agree to some extent (2) I disagree (1)	
	**4. My injury can cause problems for my family.**	2.552 (0.654)
	I quite agree (3) I agree to some extent (2) I disagree (1)	
	**5. My injury can cause disability. **	2.410 (0.689)
	I quite agree (3) I agree to some extent I disagree (1)	
**Perceived benefits**	**1- If I follow the safety notes, it will help me avoid injuries. **	2.708 (0.603)
	I quite agree (3) I agree to some extent (2) I disagree (1)	
	**2- If I follow the safety notes, it will keep me safe. **	2.784 (0.542)
	I quite agree (3) I agree to some extent (2) I disagree (1)	
	**3. If I take caution when crossing the street, I will not be injured.**	2.708 (0.569)
	I quite agree (3)	
	I agree to some extent (2)	
	I disagree (1)	
	**4. If I follow the safety notes, it will prevent problems happening to my family **	2.590 (0.653)
	I quite agree (3)	
	I agree to some extent	
	I disagree (1)	
**Perceived barriers**	**1- I do not have enough time to always cross from the crosswalk. **	2.456 (0.714)
	I quite agree (3)	
	I agree to some extent (2)	
	I disagree (1)	
	**2- It's hard for me to only cross from the crosswalk. **	2.570 (0.700)
	I quite agree (3) I agree to some extent (2) I disagree (1)	
	**3. Because the footbridge has many stairs and it is high, it is difficult for me to cross it.**	2.584 (0.675)
	I quite agree (3)	
	I agree to some extent (2)	
	I disagree (1)	
	**4. It's hard for me to fasten the seatbelt because it makes me feel annoyed.**	2.524 (0.714)
	I quite agree (3)	
	I agree to some extent (2)	
	I disagree (1)	
	**5. I like to sit in the front seat of the car because it's boring to sit on the backseat.**	2.480 (0.737)
	I quite agree (3)	
	I agree to some extent (2)	
	I disagree (1)	
	**6. Using of helmet makes me unable to see my surroundings well. **	2.590 (0.677)
	I quite agree (3) I agree to some extent (2)	
	I disagree (1)	
	**7. It's hard for me to use a helmet because it makes me feel hot. **	2.508 (0.704)
	I quite agree (3)	
	I agree to some extent (2)	
	I disagree (1)	
**Self-efficacy**	**1- I can take road safety tips when crossing the street. **	2.786 (0.507)
	I quite agree (3) I agree to some extent (2) I disagree (1)	
	**2. I can take safety tips while getting on a car. **	2.732 (0.552)
	I quite agree (3) I agree to some extent (2) I disagree (1)	
	**3. I can take safety tips while riding a motorcycle. **	2.744 (0.561)
	I quite agree (3) I agree to some extent (2) I disagree (1)	
	**4. I can take safety tips while riding a bike. **	2.688 (0.599)
	I quite agree (3) I agree to some extent (2) I disagree (1)	

**Appendix 2:** Checklist to observe the students' road-crossing-behaviors

Road-crossing behaviors	Results of the observations	
	
1. Did the student cross the street using crosswalk?	Yes	No
2. Did the student cross the street when the traffic light was green?	Yes	No
3. Did the student stop and wait for the light to turn green when the traffic light was red?	Yes	No
4. Did the student first look at the left side when crossing the street?	Yes	No
5. Did the student stop in the middle of the street when crossing the street and then look at the right side?	Yes	No

**Data analysis**

Regarding the scores of knowledge, performance and constructs of the model, the score of less than 50 is weak, the score between 50 to 75 is average and the score greater than 75 is desirable. Collected data were analyzed using SPSS 16. For all variables, descriptive statistics were calculated and also to find independent variable, the multiple linear regression with backward elimination method was used for self-reported behavior and also observed behavior, separately. The levels of 0.05 and 0.1 were considered for entry-level and removing variables in the model, respectively.

## Results

Out of 500 students participating in this study, half of them were in the fourth grade and the others were in the fifth grade. The mean age of them was 10.51±0.50. 50.6% and 49.4% of them were boys and girls, respectively. The mean of their household size was 4.43±1.106.

Table 1 shows the demographic data. The greatest level of fathers’ education was more than high school diploma (37.8%) and 37.6% of mothers’ education level was lower than high school diploma. About occupation, 41.6% of fathers were privately paid and 75% of mothers were housewives. Most of the students walked to school with their friends (41%). 83.4% of the students’ fathers had a type of vehicles. 

**Table 1:** Distribution of demographic characteristics of the participants. (n=500)

Characteristics	Number	Percentage
**The students’ sex**		
Male	253	50.6
Female	247	49.4
**Students’ grade**		
4	250	50
5	250	50
**Mothers’ education**		
<High school diploma	188	37.6
High school diploma	144	28.8
>High school diploma	168	33.6
**Fathers’ education**		
<High school diploma	183	36.6
High school diploma	128	25.6
>High school diploma	189	37.8
**Mothers’ occupation**		
Housewife	375	75
Employee	91	18.2
Private working	14	2.8
Retired	11	2.2
Other	8	1.6
**Fathers’ occupation**		
Laborer	83	16.6
Employee	153	30.6
Private working	208	41.6
Retired	27	5.4
Other	29	5.8
**Does your father have any types of vehicles?**		
Yes	417	83.4
No	83	16.6
**How do you go to school and come back home?**		
On foot, with adults	31	6.2
By vehicle, with adults	69	13.8
On foot, with friend(s)	205	41
On foot, alone	53	10.6
With school bus	132	26.4
Other	10	2

According to Table 2 , the results of HBM constructs show that the mean scores of knowledge, perceived susceptibility, perceived severity, perceived benefit, perceived barrier and self-efficacy were 64.139, 82.817, 82.453, 89.917, 84.343 and 91.250, respectively. The mean score of the students’ self-reported performances about traffic injury prevention was 48.750 and the mean score of their observed road-crossing behavior in the simulated street was 45.000. In this study, the rate of using helmet while riding a bike and motorcycle was 25.8% and 23.6%, respectively, and 53.4% of the students fastened the seatbelt when getting on a motor vehicle. Regarding the cues for action, the students reported that they had been trained about different aspects of traffic instructions mostly by their parents (including red light 41.6%, crossing the street 58.2%, getting on the car 64.4% riding the motorcycle 49.4%, riding the bike 64.4%, sitting on the backseat of the car 64.4%), while the police were the second reported source of traffic information by them. When the students were asked about by whom they would like to be trained, more than half of them (55%) answered they would prefer to learn from their parents. 

**Table 2:** The mean scores of students' self-reported performances, knowledge, observed road-crossing behaviors and HBM constructs.

Variable	Mean *	SD**
Student' self-reported performances	48.750	20.690
Knowledge	64.139	19.011
Perceived susceptibility	82.817	15.177
Perceived severity	82.453	16.303
Perceived benefits	89.917	15.056
Perceived barriers	84.343	16.472
Self-efficacy	91.250	15.032
Observed road-crossing behaviors	45.000	22.108

*The mean of total score, calculated out of 100 points. **Standard deviation

Table 3 shows the final model of multiple linear regressions with backward elimination. In the final model, it was demonstrated that the students’ sex, knowledge, perceived susceptibility, self-efficacy, and observed road-crossing behavior in the simulated street predict the students’ self-reported injury prevention performance. These variables, all together determine 17.6% of total variation of the self-reported preventive performances.

**Table 3:** All steps of significant variables on students' self-reported performances, using multiple linear regression models.

Step	Variable	Beta	Standard Error	Standard Beta	t-value	P-value	r	R2
Step 1	Sex (Male=0)	4.231	1.843	0.102	2.296	0.022	0.102	0.010
Step 2	Sex(Male=0)	5.265	1.758	0.127	2.995	0.003	0.328	0.107
	Knowledge	0.340	0.046	0.312	7.341	<0.001		
Step 3	Sex(Male=0)	5.947	1.739	0.144	3.419	0.001	0.369	0.136
	Knowledge	0.293	0.047	0.269	6.223	<0.001		
	Perceived susceptibility	0.239	0.059	0.269	4.050	<0.001		
Step 4	Sex(Male=0)	5.301	1.745	0.128	3.037	0.003	0.385	0.148
	Knowledge	0.273	0.047	0.251	5.777	<0.001		
	Perceived susceptibility	0.212	0.060	0.156	3.558	<0.001		
	Perceived barriers	0.145	0.054	0.116	2.678	0.008		
Step 5	Sex(Male=0)	5.376	1.728	0.128	3.111	0.002	0.408	0.167
	Knowledge	0.259	0.047	0.238	5.502	0.000		
	Perceived susceptibility	0.172	0.060	0.126	2.854	0.004		
	Perceived barriers	0.115	0.054	0.091	2.102	0.036		
	Self-efficacy	0.093	0.040	0.100	2.319	0.001		
Step 6	Sex(Male=0)	5.568	1.722	0.135	3.233	0.001	0.419	0.176
	Knowledge	0.225	0.049	0.207	.6074	<.001		
	Perceived susceptibility	0.184	0.060	0.135	.0623	0.002		
	Perceived barriers	0.116	0.054	0.093	2.145	0.032		
	Self-efficacy	1.692	0.497	0.147	3.404	0.001		
	Observed road-crossing behaviors	0.094	0.040	0.101	2.358	0.019		

According to Table 4 , in the final model of multiple linear regressions, the variables of self-reported behavior, knowledge and perceived susceptibility predict the students’ observed behavior. These variables all together determine about 9.2% of total variation of the observed road-crossing behavior.

**Table 4:** All steps of significant variables on students' observed road-crossing behaviors, using multiple linear regression models.

Step	Variable	Beta	Standard Error	Standard Beta	t-value	P-value	r	R2
Step 1	Self-reported performances	0.158	0.047	0.148	3.337	0.001	0.148	0.022
Step 2	Self-reported performances	0.076	0.048	0.071	1.569	0.117		
	Knowledge	0.297	0.052	0.255	5.653	<0.001	0.284	0.081
Step 3	Self-reported performances	0.095	0.049	0.089	1.956	0.051	0.303	0.092
	Knowledge	0.322	0.053	0.277	6.055	<0.001		
	Perceived susceptibility	-0.160	0.065	-0.110	-2.447	0.015		

## Discussion

In this study, most of the students walked to school with their friends. The most of the students' fathers had vehicles. The average scores of students’ knowledge, performances and both self-reported and observed behaviors were not at desirable levels. There was a significant relationship between performance, sex, knowledge, perceived susceptibility, perceived barriers, self-efficacy, and observed road-crossing behavior in the simulated street. 

The study on factors related injuries among children, conducted in west of Iran showed a statistically significant correlation between mothers’ knowledge and injury severity among children.^19^ This finding shows the importance of increasing awareness to help injury prevention among studied population. In Nazari et al.’s study, the average scores of knowledge and students’ performance were not at desirable levels and these findings are similar to results of current study.^20^ It seems that lack of necessary road and pedestrian safety training and inefficacy of non-systematic education in the community and among students can be one of the main reasons.

The results of cues to action showed that the participants had received some instructions about road traffic and pedestrian safety firstly from their parents and then from the police and their teachers, respectively. The results of two studies, one was conducted in the U.S.A by Barton et al. (2004) and another was conducted in Australia, by Axley et al. (2006), showed that most parents instructed their children about crossing the street. Therefore, the behavioral role of parents, the police and teachers must be considered in designing an injury prevention program.^21,22^

In Mehri et al.’s study, in Sabzevar, in Iran, the most important persons who encouraged participants to fasten the seatbelt were family members, the police, friends and colleagues.^23^ In current study, 25.8% of the students reported that they wore helmet while riding a motorcycle and 23.6% of them wore helmet while riding a bike. In spite of growing people’s knowledge in some cases, the rate of doing the safe behaviors such as wearing helmet is relatively low in the study setting. The reason may be not having positive attitude towards these behaviors and/or not being comfortable when wearing helmet. The results of Hung et al.’s study in Vietnam, Li in China and Bianco in Italy were about investigating barriers of wearing helmet and they focused on the above mentioned points.^24,25,26^ The results of Orouji et al.’s study in Khomein and Baghianimoghadam et al.'s in Yazd, both in Iran, and were about participants' attitude and injury protective behaviors confirm the findings of this study.^27,28^

In this study, 53.4% of the students reported that they fastened seatbelt while getting on the car. The results of Elvik & Christen’s study showed that the obligation to fasten seatbelt increased the rate of fastening seatbelt. Therefore, obligation imposed by the police could be considered as an intervention and increased the use of seatbelt.^29^

The results of multiple linear regressions showed that there was a significant relation between injury prevention performances and, sex, knowledge, perceived susceptibility, perceived barriers, self-efficacy and road-crossing behaviors while there was no significant relation between performances and perceived benefits as Mehri et al. also showed in their study.^23^ In a study, conducted in Khomein, Iran by Orouji et al. there was a significant relation between performance and perceived susceptibility, barriers, benefits, and cues to action.^27^ It is compatible with other studies like Servadei et al.’s in Italy which showed that the perceived benefits and barriers were the most important factors in performance such as using helmet while riding a bike.^30^ In our study, performance had a significant relation with knowledge and Dong X et al. study in China confirms this result. ^31^ In Lawrence et al.’s study in Australia, it is showed that parents’ attitude towards road risks had significant relationwith road-crossing behavior while there was no significant relation between performance and knowledge regarding to road risks.^32^ Holakui et al. showed that in their study there was no statistically significant relation between pedestrians’ performance and their awareness and that is not compatible with this study.^14^ Therefore, traffic regulations must be strongly enforced to improve the road traffic injury preventive behaviors.^14^

In this study, the average score of road-crossing behaviors in the simulated street was 45. Only 61.2% of the participants walked across that street. 72% of them crossed the street with green light and 39.6% with red light while 59.8% of them stopped with red light. 27.4% of the participants checked their left side and 72% of them checked their right side while starting to cross the street. 3.2% of the participants stopped in the middle of the street and then looked at their right side and 96.2% of them crossed the street without stopping. The results of Khan et al.’s study in Karachi, Pakistan, showed that 60% of participants looked at their left or right side while crossing the street, 47% of them crossed the street without stopping and 77% of them crossed the parts of the street which were not crosswalks.^33^ Due to limitation of self-report method, we observed the students’ behaviors in a simulated street in the school, using observation checklist.

In current study, the final multiple linear regression predict the injury preventive performances, although the prediction power of the dependent variables by independent variables was poor and was about 18%. It should be noted that the main aim of this study was not prediction. Instead, it was to determine the factors related to injury preventive behaviors among students, which can be for designing any effective program. 

Generally, this study showed that students’ performances, regarding to prevent RTIs in this study setting was undesirable. For designing and implementing an educational program, we can collaborate with parents, the police and teachers. For appropriate educational programs, it is necessary to ascertain the individuals’ position and educational needs in relation to knowledge, attitude and performance. To design any educational programs, it is also necessary to have information about vulnerable groups of people. The longitudinal study and intervention programs can be applied for all of social groups to promote safety and prevent injuries among them.

## Conclusion

The results of this study show that the scores of knowledge and injury prevention performances are not at desirable levels among studied students. Also, the results of multiple linear regressions show that there is a significant relation between injury prevention performances and sex, knowledge, perceived susceptibility, perceived barriers, self-efficacy and the road-crossing behaviors among students. Regarding the undesirable level of knowledge and low level of injury prevention performances among studied students, it is necessary to provide appropriate traffic safety programs for them while parents, the police and teachers must be involved in those programs. HBM constructs focus mostly on individual beliefs and they can only predict a small variation of road traffic injury prevention behaviors among studied population. Therefore this study confirms that in an interventional program, educating individuals must be focused on improving individuals’ skills in addition to their knowledge and attitude and also must be combined with other related interventional components including environmental and vehicle related factors in order to have a more efficient program.

**Acknowledgement **

This study has been approved by the Deputy of Research and Technology of Hamadan University of Medical Sciences (reference number: 920127219).
